# Supplementary material for: Enhanced Brain‐Heart Connectivity as a Precursor of Reduced State Anxiety after Therapeutic Virtual Reality Immersion
Source: Adv Sci (Weinh). 2025 Aug 11;12(38):e03606. doi: 10.1002/advs.202503606 (PMC12520498; doi:10.1002/advs.202503606)
Supplement: Supplementary file 1 — Supporting Information [file ADVS-12-e03606-s002.docx]

**Supporting Information for**

**Enhanced Brain-Heart Connectivity as a Precursor of Reduced State Anxiety After Therapeutic Virtual Reality Immersion**

Idil Sezer^1,2,Δ^, Paul Moreau^2,3^, Mohamad El Sayed Hussein Jomaa^2^, Valérie Godefroy^4^, Bénédicte Batrancourt^1^, Richard Lévy^1,5^, Anton Filipchuk^2,Δ^

1. Paris Brain Institute, FrontLAB, CNRS INSERM U1127, Pitié-Salpêtrière Hospital, Paris, France

2. Healthy Mind, 11 rue de Lourmel, Paris, France

3. Sorbonne University, INSERM, CNRS, Vision Institute, Paris, France

4. Center for Neuroscience Research Lyon, SocialHealth lab CNRS / INSERM, Lyon, France

5. Sorbonne University, AP-HP, Institute of Memory and Alzheimer's Disease, Department of Neurology, Pitié-Salpêtrière Hospital, Paris, France

Δ Corresponding authors: Anton Filipchuk, Idil Sezer

**Emails:**

anton.filipchuk@neuromind.fr

[idil.sezer@icm-institute.org](mailto:Idil.sezer@icm-institute.org)


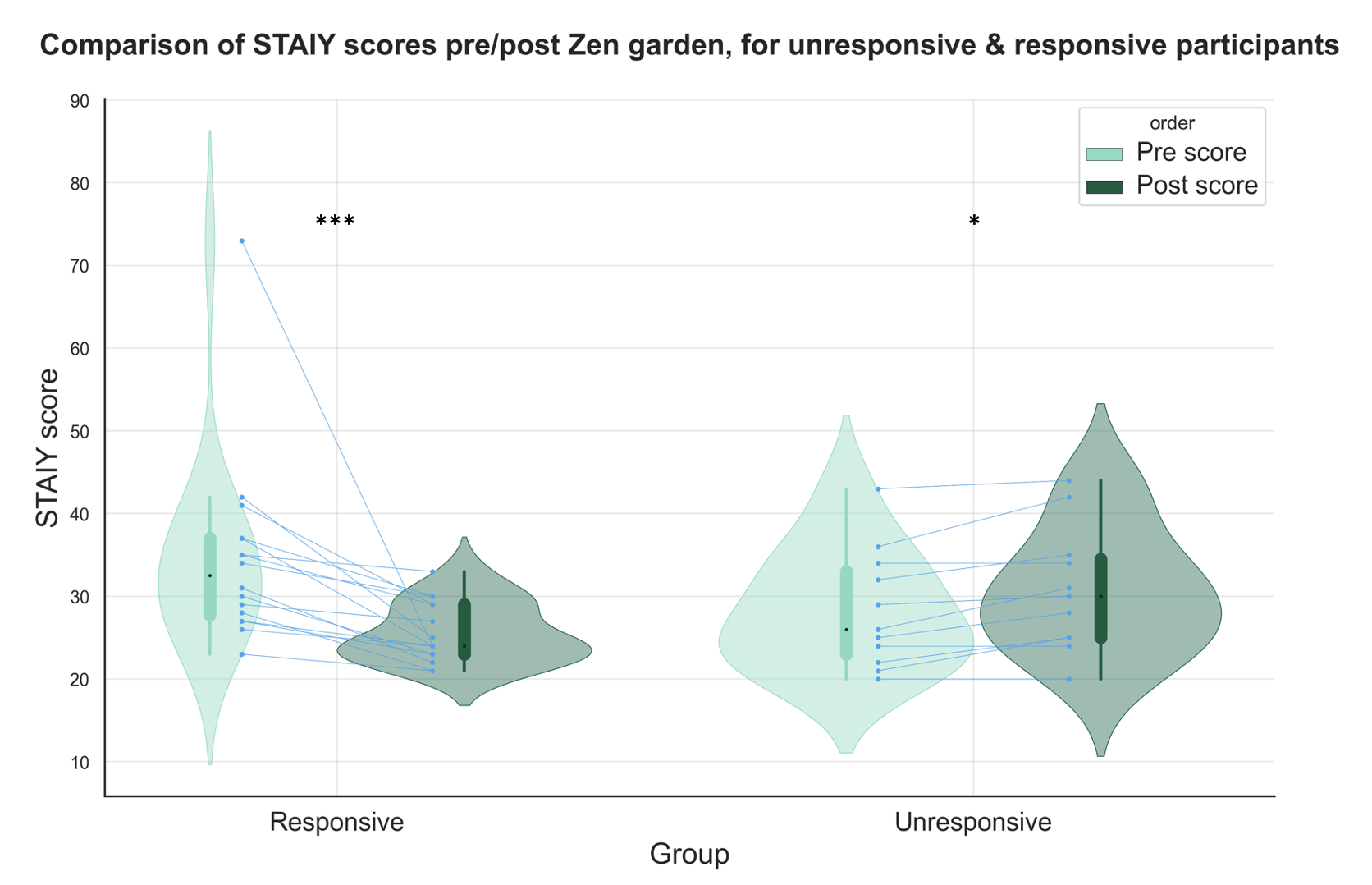
Figure S2. Zen Garden condition effects on psychometric ratings in each group. STAI-Y1 state anxiety scores of responsive (p=0.0005, *W*=136, *r*=0.87, 95% CI [0.87, 0.89]) and unresponsive (p=0.01, *W*=0, *r*=-0.74, 95% CI [-0.88, -0.57]) participant groups. Control cities and *Zen Garden* conditions depicted in bright green and dark green, respectively. Each participant’s data is represented by connecting blue points (N=16 responsive and n=11 unresponsive participants). The boxplot inside the violin plot corresponds to the interquartile range, the median is depicted with a black dot, the vertical bright and dark green lines correspond to the probability density function. Statistical analyses performed using Wilcoxon signed rank test.


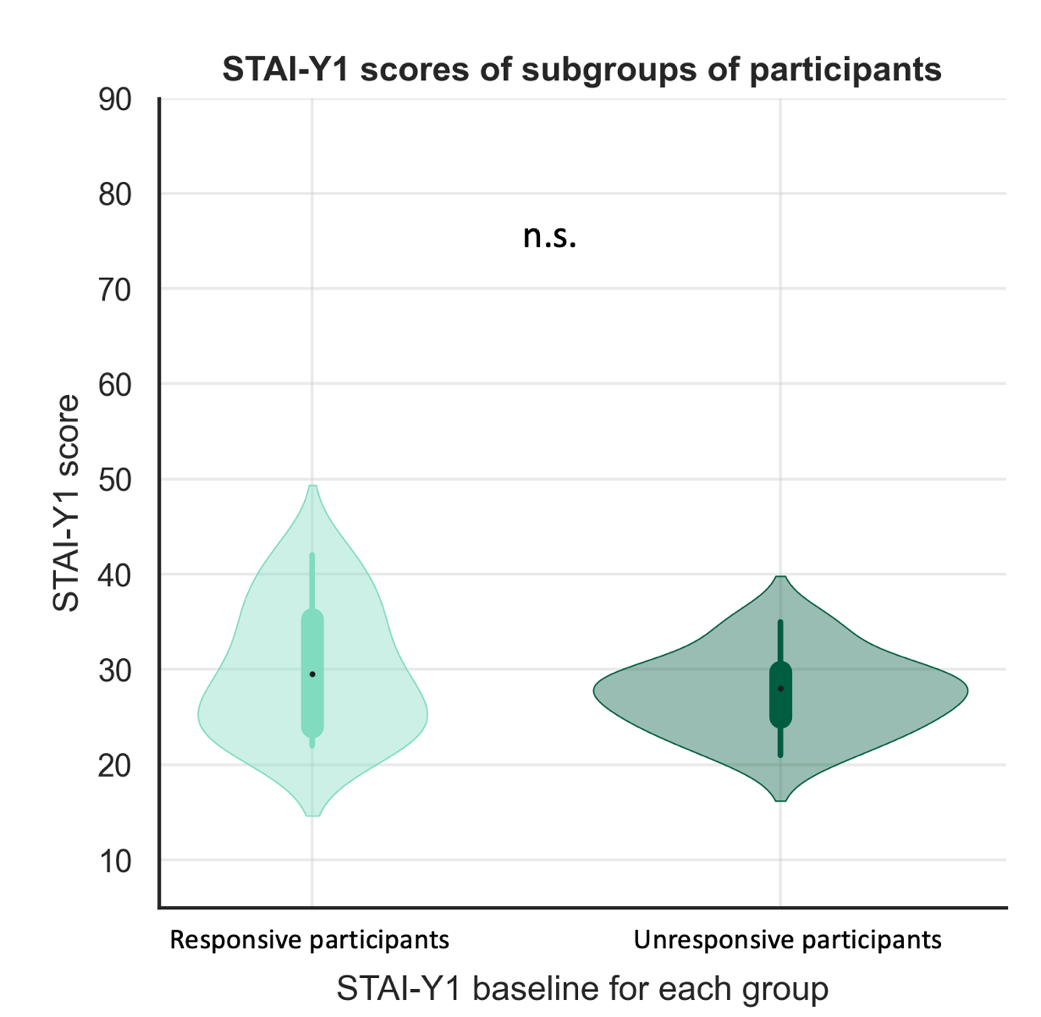


Figure S2. Baseline state anxiety levels. Baseline STAI-Y1 state anxiety scores in participants classified as responsive or unresponsive (p= 0.71), before viewing *Zen Garden* or control videos. N=16 responsive and n=11 unresponsive participants. The boxplot inside the violin plot corresponds to the interquartile range, the median is depicted with a black dot, the vertical bright and dark green lines correspond to the probability density function. Statistical analysis performed using Mann Whitney U test.


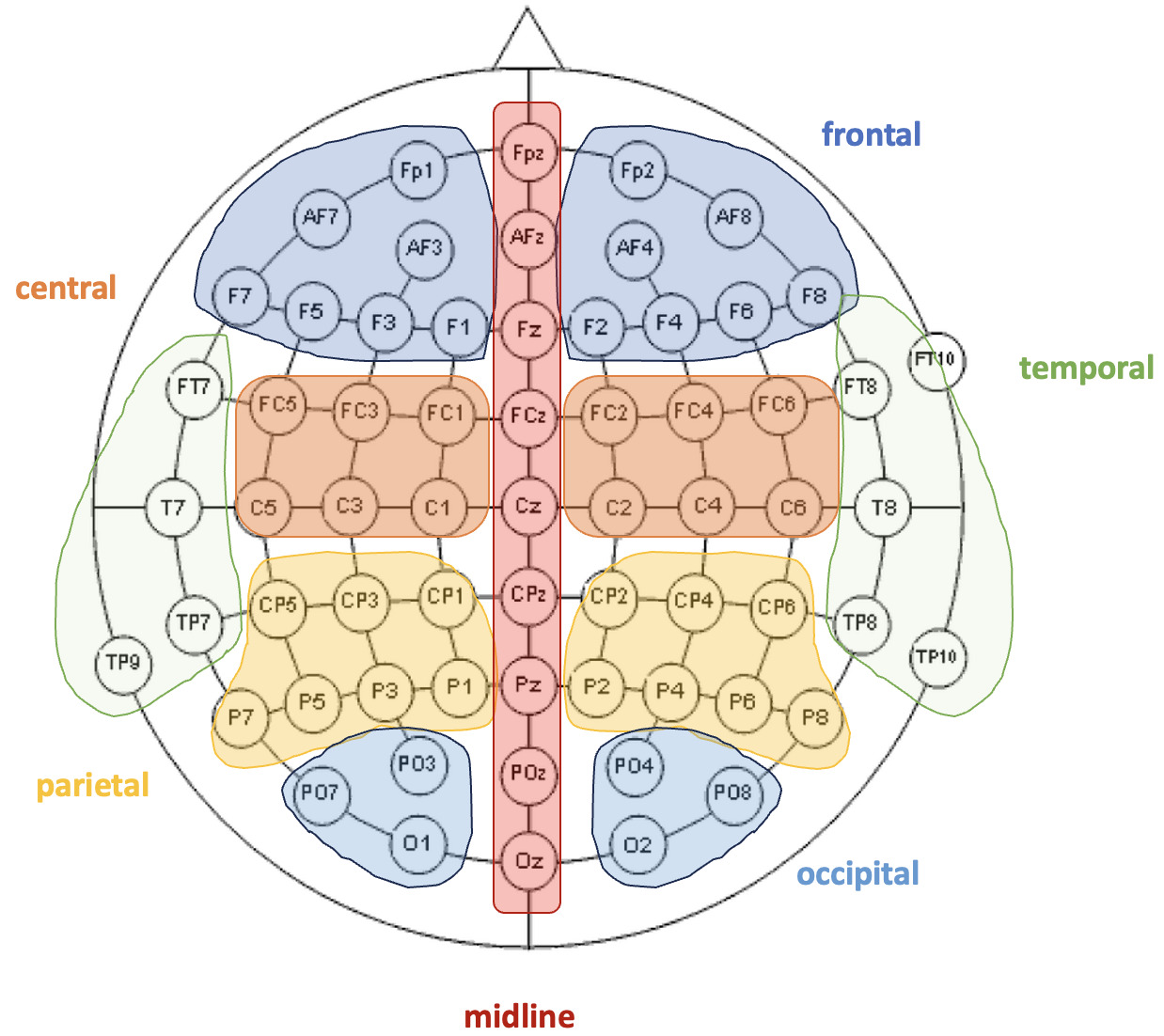


Figure S3. Grouping of electrodes by region for analyses.
Frontal region electrodes: Fp2, AF4, AF8, F8, F6, F4, F2, Fp1, AF3, AF7, F7, F5, F3, F1
Temporal region electrodes: FT8, FT10, T8, TP8, TP10, FT7, FT9, T7, TP7, TP9
Central region electrodes: FC2, FC4, FC6, C6, C4, C2, FC1, FC3, FC5, C5, C3, C1
Parietal region electrodes: CP2, CP4, CP6, P6, P4, P2, CP1, CP3, CP5, P5, P3, P1
Occipital region electrodes: P8, PO8, PO4, O2, P7, PO7, PO3, O1
Midline region electrodes: AFz, Fz, Cz, CPz, Pz, POz, Oz, Iz


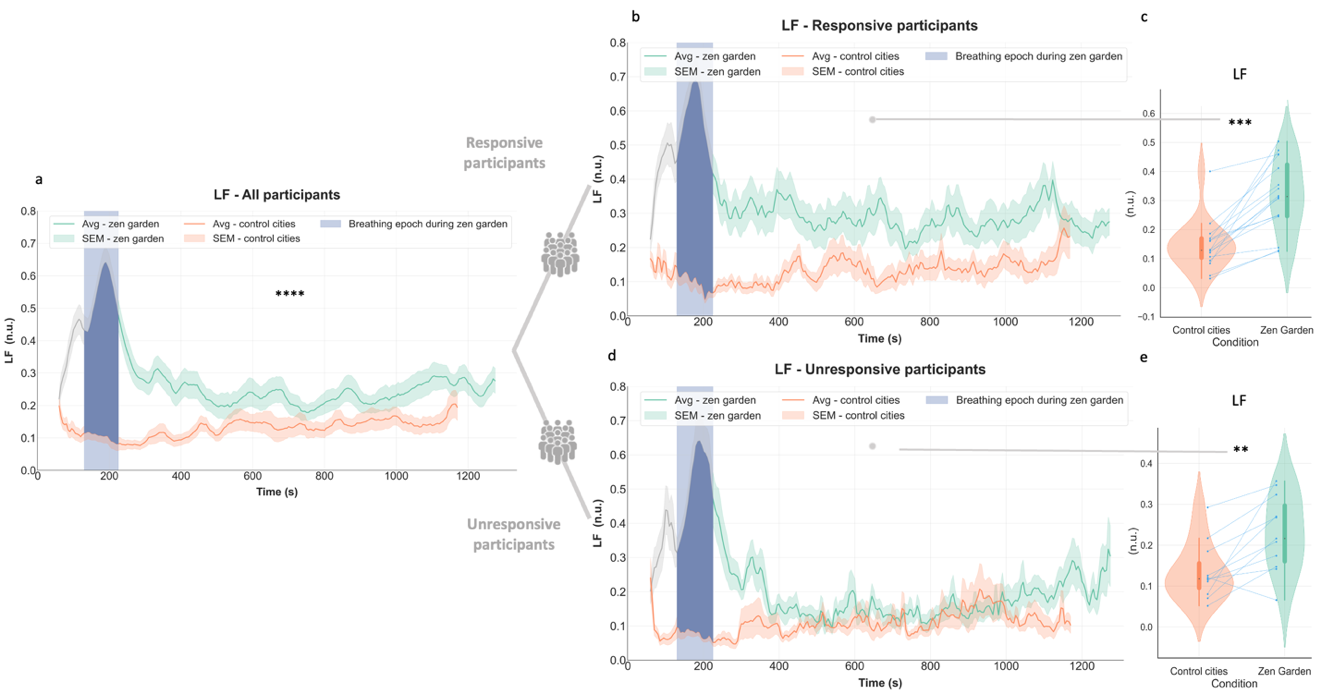


Figure S4. ECG LF spectral power response in each subgroup. a Temporal plots depict time-varying ECG LF differences across conditions in all participants (see Fig 1, d for statistics) b Responsive participants exhibited increase in LF spectral power values during the *Zen Garden* vs control conditions over 20-minute duration c Averaged LF spectral power increased during the *Zen Garden* condition (excluding the paced breathing induction epoch) compared the control video in the responsive group (p=0.0003, *W*=136, *r*=0.74, 95% CI [0.38, 0.91]) d Unresponsive participants exhibited increase in LF spectral power values during the *Zen Garden* compared to control condition over 20-minute duration e Unresponsive participants’ averaged LF spectral power also increased during the *Zen Garden* condition compared the control condition (p=0.009, *W*=63, *r*=0.59, 95% CI [0.06, 0.86]).
*Zen Garden* and control cities conditions depicted in green and orange, respectively. Temporal plots (b,d) depict data points for each window size=1 minute and step size=5 seconds, averaged ± SEM. Removed breathing exercise epoch is highlighted in blue. For (c,e) each participant’s data is represented by connecting blue points. The boxplot inside the violin plot corresponds to the interquartile range, the median is depicted with a black dot, the vertical green and orange lines correspond to the probability density function. N=27 all participants, n=16 responsive and n=11 unresponsive participants. Wilcoxon signed rank test, adjusted with False Discovery Rate correction.


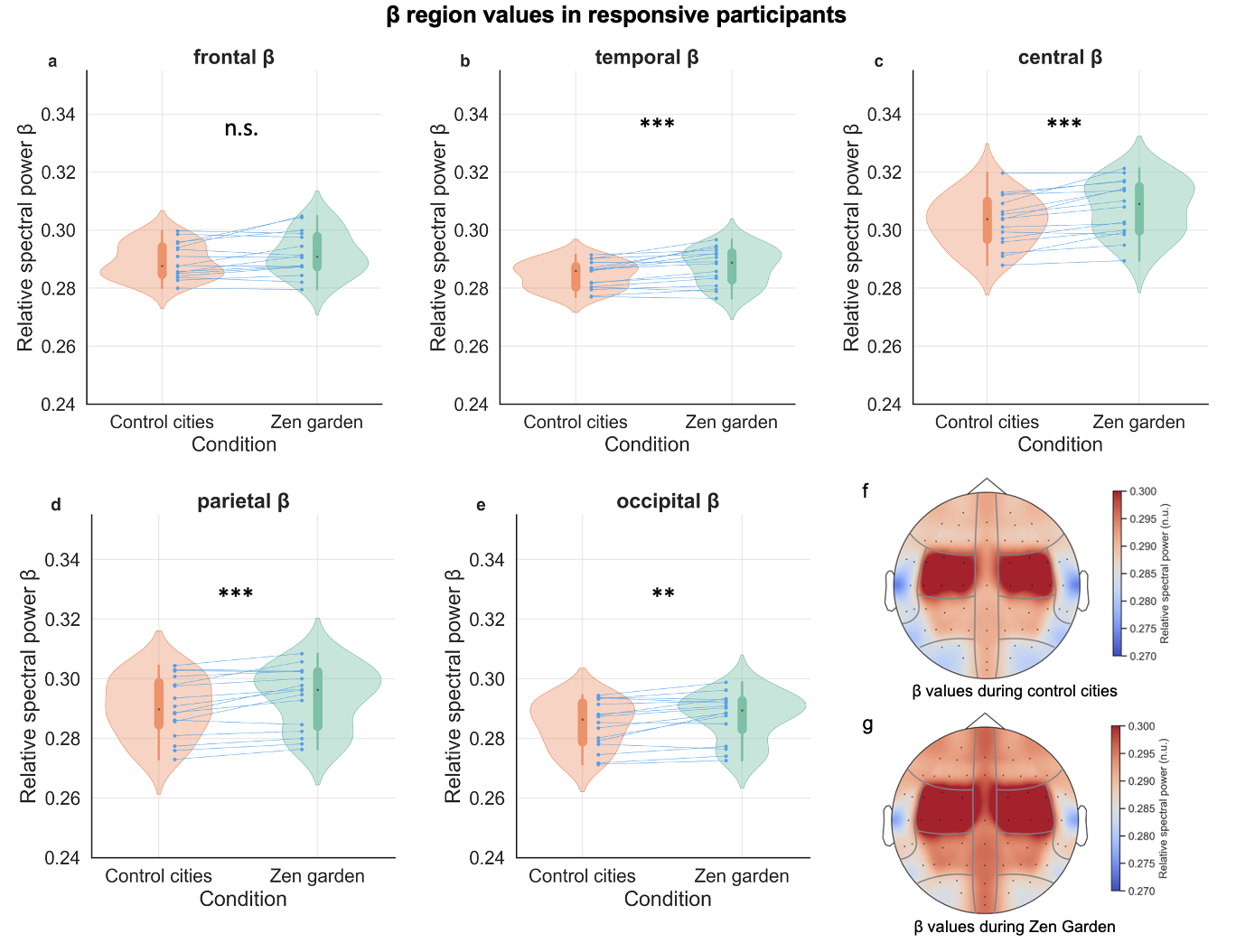


Figure S5. Conditions effects on beta regions in responsive participants. a,b,c,d,e Beta relative spectral power (13-30 Hz) variations between *Zen Garden* and control video conditions in responsive participants in a frontal (p=0.07), b temporal (p=0.001, *W*=130, *r*=0.30, CI [-0.12, 0.64), c central (p=0.0009, W=134, *r*=0.28, CI [-0.14,0.62]), d parietal (p=0.001, *W*=130, *r*=0.17 [-0.24, 0.53]), e occipital (p=0.006, *W*=122, *r*=0.18, CI [-0.24, 0.54]) regions. Across conditions, temporal, central parietal and occipital regions displayed statistically significant difference in this responsive group, whereas the frontal region did not. f,g Topographic representation illustrating averaged beta relative spectral power in responsive participants f during the control condition and g during the Zen Garden condition. The groups of electrodes are averaged as in Figure S3.
*Zen Garden* and control cities description conditions depicted in green and orange, respectively. Each participant’s data is represented by connecting blue points (N=16 participants). The boxplot inside the violin plot corresponds to the interquartile range, the median is depicted with a black dot, the vertical green and orange lines correspond to the probability density function. Wilcoxon signed rank test, adjusted with False Discovery Rate correction.


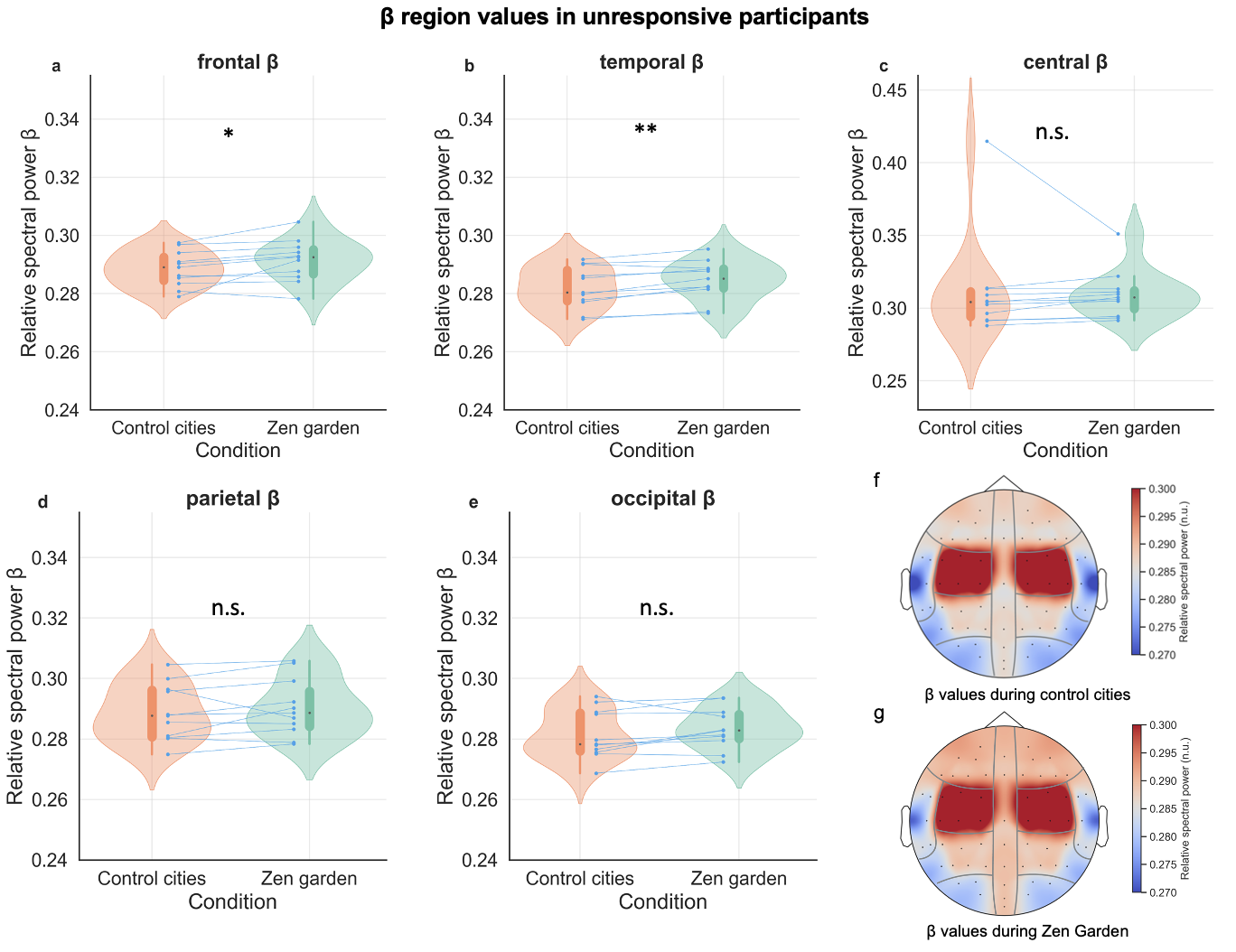


Figure S6. Conditions effects on beta regions in unresponsive participants. a,b,c,d,e Beta relative spectral power (13-30 Hz) variations between *Zen Garden* and control video conditions in unresponsive participants in a frontal (p=0.04, *W*=58, *r*=0.26, CI [-0.26, 0.66]), b temporal (p=0.006, *W*=64, *r*=0.21 [-0.30, 0.62]), c central (p=0.07) , d parietal (p=0.14), e occipital (p=0.07) regions. Across conditions, the frontal and temporal regions displayed statistically significant difference in this unresponsive group. f,g Topographic representation illustrating averaged beta relative spectral power in unresponsive participants f during the control condition and g during the *Zen Garden* condition. The groups of electrodes are averaged as in Figure S3.
*Zen Garden* and control cities description conditions depicted in green and orange, respectively. Each participant’s data is represented by connecting blue points (N=11 participants). The boxplot inside the violin plot corresponds to the interquartile range, the median is depicted with a black dot, the vertical green and orange lines correspond to the probability density function. Wilcoxon signed rank test, adjusted with False Discovery Rate correction.


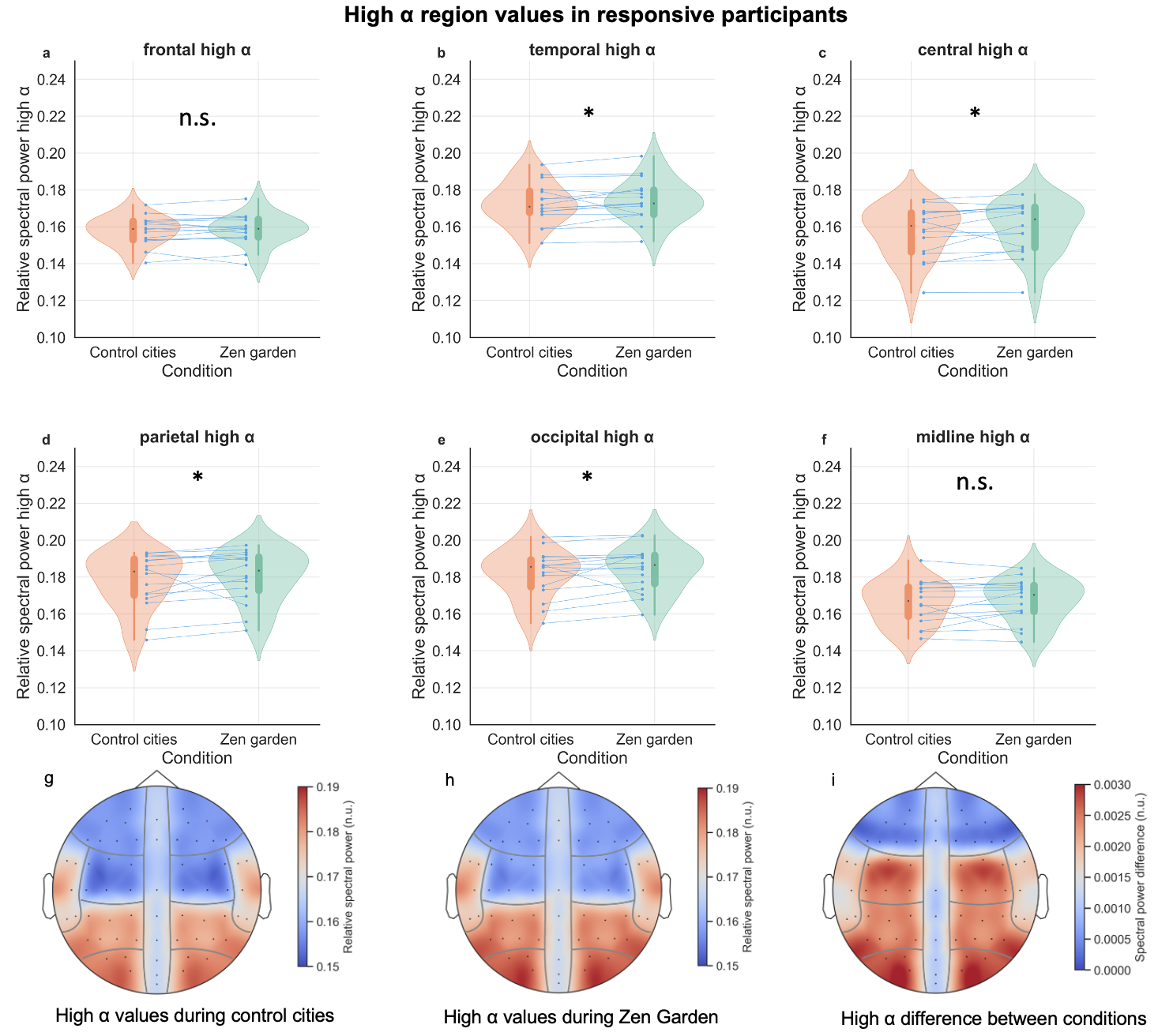


Figure S7. Supplementary Figure 7. Conditions effects on high alpha regions in responsive participants. a,b,c,d,e,f High alpha relative spectral power (10-13 Hz) variations between *Zen Garden* and control video conditions in responsive participants in a frontal (p=0.49), b temporal (p=0.04, *W*=112, *r*=0.11, CI [-0.30, 0.48]), c central (p=0.04, *W*=110, *r*=0.16, CI [-0.29, 0.52]), d parietal (p=0.04, *W*=112, *r*=0.12, CI [-0.28,0.49]), e occipital (p=0.04, *W*=116, *r*=0.16, CI [-0.26,0.52]) and f midline (p=0.30) regions. Across conditions, temporal, central, parietal and occipital regions displayed statistically significant difference in this responsive group, whereas the frontal region did not. g,h,i Topographic representation illustrating averaged high alpha relative spectral power in responsive participants g during the control condition and h during the *Zen Garden* condition, i contrast between conditions (*Zen Garden* – control conditions). The groups of electrodes are averaged as in Figure S3.
*Zen Garden* and control cities description conditions depicted in green and orange, respectively. Each participant’s data is represented by connecting blue points (N=16 participants). The boxplot inside the violin plot corresponds to the interquartile range, the median is depicted with a black dot, the vertical green and orange lines correspond to the probability density function. Wilcoxon signed rank test, adjusted with False Discovery Rate correction.


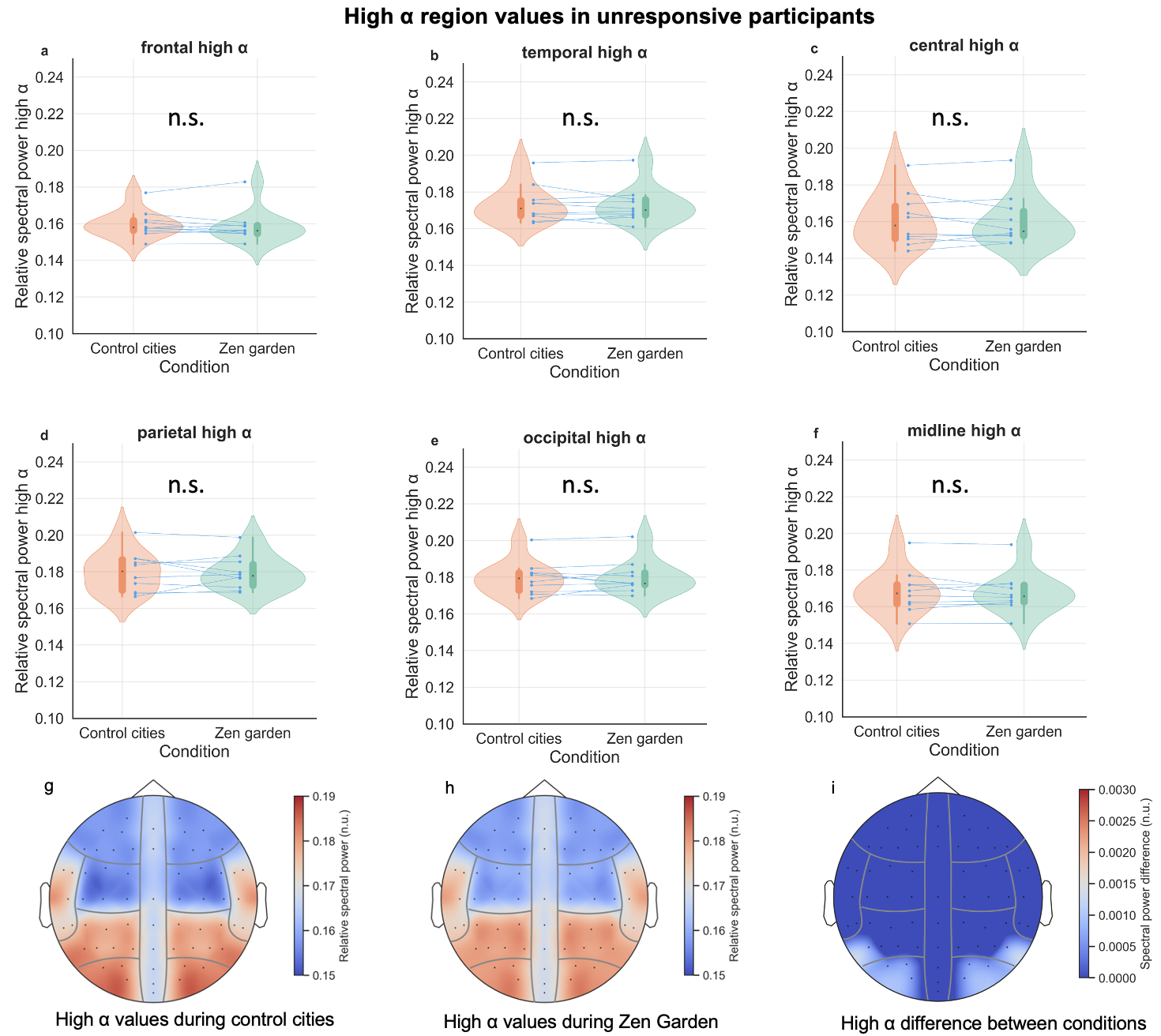


Figure S8. Conditions effects on high alpha regions in unresponsive participants. a,b,c,d,e,f High alpha relative spectral power (10-13 Hz) variations between *Zen Garden* and control video conditions in unresponsive participants in a frontal (p=1), b temporal (p=1), c central (p=1), d parietal (p=1), e occipital (p=1) and f midline (p=1) regions. g,h,i Topographic representation illustrating averaged high alpha relative spectral power in unresponsive participants g during the control condition and h during the *Zen Garden* condition, i contrast between conditions (*Zen Garden* – control conditions). Wilcoxon signed rank test, adjusted with False Discovery Rate correction. The groups of electrodes are averaged as in Figure S3.
*Zen Garden* and control cities description conditions depicted in green and orange, respectively. Each participant’s data is represented by connecting blue points. N=11 participants, one participant was excluded after identifying outliers based on the 5%-95% range calculation, resulting in n=10 participants. The boxplot inside the violin plot corresponds to the interquartile range, the median is depicted with a black dot, the vertical green and orange lines correspond to the probability density function. Wilcoxon signed rank test, adjusted with False Discovery Rate correction.

**
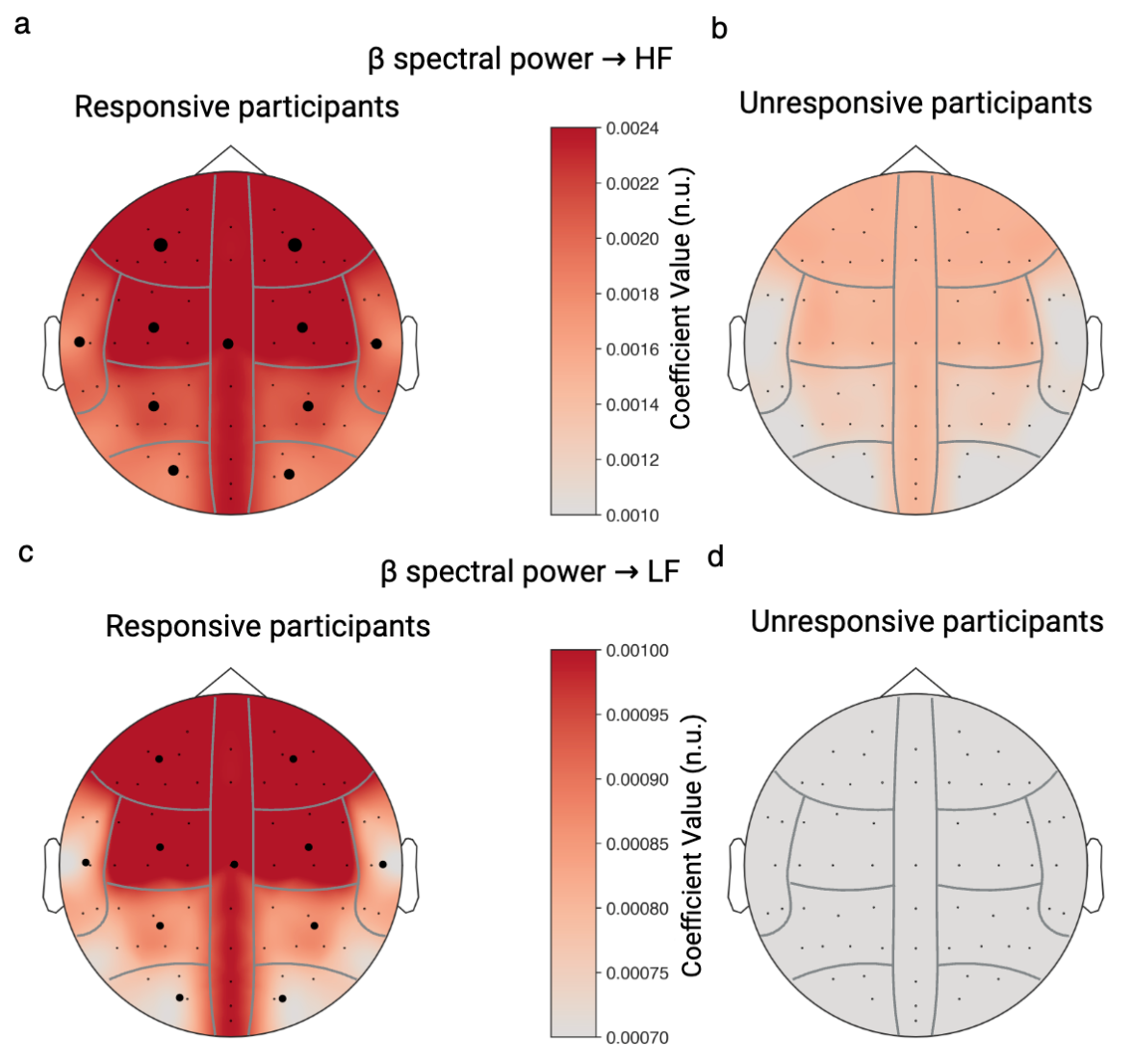
**

**Figure S9. Conditions effects on brain to heart connectivity coefficients in responsive and unresponsive participants. a, b** Topographic representation illustrating averaged beta spectral power to high frequency (B2HF) coefficient values across regions in **a** responsive and **b** unresponsive participants, contrast between conditions (*Zen Garden* – control conditions). **a** In responsive participants B2HF coefficient values differ significantly between conditions in frontal (p=0.0009, W=3, r=–0.33, CI [–0.652, 0.098]), central (p=0.0025, W=9, r=–0.27, CI [–0.611, 0.148]), midline (p=0.0025, W=10, r=–0.27, CI [–0.603, 0.152]), parietal (p=0.0032, W=13, r=–0.27, CI [–0.611, 0.148]), temporal (p=0.0025, W=11, r=–0.33, CI [–0.654, 0.100]) and occipital (p=0.0034, W=14, r=–0.30, CI [–0.631, 0.130]) regions. **b** In unresponsive participants, no significant differences are observed in B2HF coefficient values across regions: frontal (p=0.16), central (p=0.32), midline (p=0.38), parietal (p=0.43), temporal (p=0.23), occipital (p=0.38).

**c, d** Topographic representation illustrating averaged beta spectral power to low frequency (B2LF) coefficient values across regions in **c** responsive and **d** unresponsive participants, contrast between conditions (*Zen Garden* – control conditions). **c** In the responsive group, B2LF coefficients differ significantly between conditions in frontal (p=0.01, W=16, r=–0.30, CI [–0.626, 0.121]), central (p=0.01, W=18, r=–0.27, CI [–0.605, 0.155]), midline (p=0.01, W=18, r=–0.27, CI [–0.605, 0.156]), parietal (p=0.01, W=19, r=–0.26, CI [–0.599, 0.163]), temporal (p=0.01, W=18, r=–0.27, CI [–0.609, 0.146]), occipital (p=0.01, W=20, r=–0.23, CI [–0.582, 0.185]) regions’. **d** B2LF coefficient values in each region did not differ between conditions in unresponsive participants: central (p=0.49), frontal (p=0.49), midline (p=0.49), occipital (p=0.49), parietal (p=0.49), temporal (p=0.43). Wilcoxon signed rank test, adjusted with False Discovery Rate correction. Statistically significant regions are marked with black dots, where larger dots indicate smaller p-values (i.e., stronger statistical significance). The groups of electrodes are averaged as in Figure S3.

**
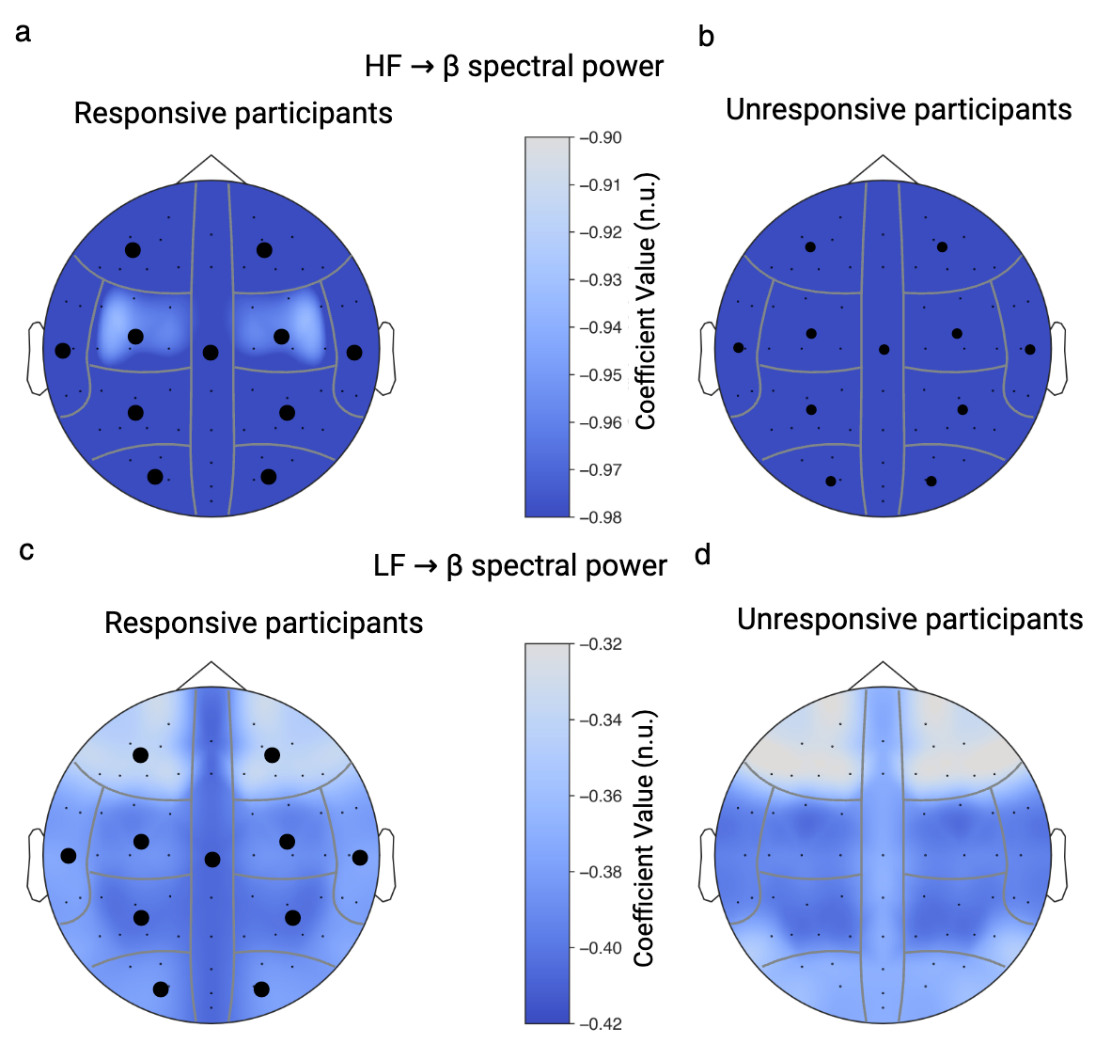
**

**Figure S10. Conditions effects on heart to brain connectivity coefficients in responsive and unresponsive participants. a, b** Topographic representation illustrating averaged high frequency to beta spectral power (HF2B) coefficient values across regions in **a** responsive and **b** unresponsive participants, contrast between conditions (*Zen Garden* – control conditions). **a** In responsive participants, HF2B coefficients vary across condition in all frontal (p=0.00003, W=136, r=0.45, CI [0.036, 0.736]), central (p=0.00003, W=136, r=0.46, CI [0.046, 0.740]), midline (p=0.00003, W=136, r=0.47, CI [0.055, 0.745]), temporal (p=0.00003, W=136, r=0.46, CI [0.047, 0.740]), parietal (p=0.00003, W=136, r=0.45, CI [0.037, 0.735]), and occipital (p=0.00003, W=136, r=0.48, CI [0.062, 0.751]) regions. **b** In unresponsive participants, HF2B coefficient values differ across conditions in frontal (p=0.002, W=55, r=0.28, CI [–0.263, 0.688]), central (p=0.002, W=55, r=0.28, CI [–0.263, 0.688]), midline (p=0.002, W=55, r=0.28, CI [–0.263, 0.688]), temporal (p=0.002, W=55, r=0.26, CI [–0.276, 0.673]), parietal (p=0.002, W=55, r=0.28, CI [–0.263, 0.688]) and occipital (p=0.002, W=55, r=0.28, CI [–0.263, 0.688]) regions.

**c, d** Topographic representation illustrating averaged low frequency to beta spectral power (LF2B) coefficient values across regions in **c** responsive and **d** unresponsive participants, contrast between conditions (*Zen Garden* – control conditions). **c** In the responsive group, LF2B values changed significantly in frontal (p=0.00021, W=132, r=0.58, CI [0.130, 0.830]), central (p=0.00004, W=136, r=0.59, CI [0.149, 0.839]), midline (p=0.00004, W=136, r=0.59, CI [0.149, 0.839]), temporal (p=0.00004, W=136, r=0.56, CI [0.125, 0.817]), parietal (p=0.00004, W=136, r=0.59, CI [0.149, 0.839]) and occipital (p=0.00004, W=136, r=0.58, CI [0.129, 0.830]) regions. **d** LF2B coefficient values did not differ between conditions in unresponsive participants: central (p=0.064), frontal (p=0.064), midline (p=0.064), occipital (p=0.064), parietal (p=0.064), temporal (p=0.064).

Wilcoxon signed rank test, adjusted with False Discovery Rate correction. Statistically significant regions are marked with black dots, where larger dots indicate smaller p-values (i.e., stronger statistical significance). The groups of electrodes are averaged as in Figure S3.

**Reliability of findings**

To assess the replicability of our findings, we conducted reliability analyses for all novel results reported in the main manuscript. As the study followed a randomized, within-subject design with each participant serving as their own control, data points from the Zen Garden and control cities conditions were segmented into two halves based on an odd–even temporal point split. This approach accounts for potential temporal fluctuations in physiological signals while preserving independence between subsets.

Normality of each segment was assessed using the Shapiro–Wilk test, and based on all the results, a non-parametric Spearman’s test was applied. For each physiological metric, the correlation coefficient and associated *p*-value were calculated between the odd and even segments. Subsequently, the Spearman–Brown prediction formula was employed to estimate the reliability of the measures derived from the split-half data. The formula is expressed as:

$$rtt= \frac{2 rh}{1+rh}$$

where rtt is the predicted reliability of the entire test, and rh is the correlation coefficient between the two halves. This approach is used to assess internal consistency when test-retest reliability is not feasible within the experimental design. These analyses were performed on data from all participants, as well as separately within the responsive and unresponsive groups. All analyses were performed in Python.

Tables 1 and 2 summarize the results of the reliability analyses for the Zen Garden and control cities conditions, respectively, including normality test outcomes, correlation methods, correlation coefficients, *p*-values, estimated reliability scores, and the number of samples per segment.

In the Zen Garden condition, physiological metrics demonstrated reliability ranging from moderate (for one metric) to high and very high, with estimated coefficients between 0.63 and 0.998 for all participants. Results were similar for responsive (0.61 to 0.997) and non-responsive (0.66 to 0.998) participants.

In the Control Cities condition, reliability similarly ranged from moderate (for one metric) to high and very high (0.61 to 0.997) in all participants. Group-level analyses showed comparable findings: 0.58 to 0.998 for responsive participants and 0.64 to 0.999 for unresponsive participants.

**Table S1. Statistical summary of reliability calculations for the Zen Garden condition.**

| Physiological metric | Correlation coefficient | Correlation p-value | **Estimated reliability** | Number of samples per half |
| --- | --- | --- | --- | --- |
| **Zen Garden, all participants** | | | | |
| LF | 0.89 | <0.0001 | **0.95** | 2942 |
| HF | 0.75 | <0.0001 | **0.86** | 2942 |
| LF/HF | 0.93 | <0.0001 | **0.96** | 2942 |
| EEG midline beta | 0.46 | <0.0001 | **0.63** | 14674 |
| B2HF (midline) | 0.99 | <0.0001 | **0.998** | 14026 |
| B2LF (midline) | 0.99 | <0.0001 | **0.996** | 14026 |
| HF2B (midline) | 0.97 | <0.0001 | **0.99** | 14445 |
| LF2B (midline) | 0.95 | <0.0001 | **0.97** | 14445 |
| **Zen Garden, responsive participants** | | | | |
| LF | 0.92 | <0.0001 | **0.96** | 1744 |
| HF | 0.75 | <0.0001 | **0.86** | 1744 |
| LF/HF | 0.94 | <0.0001 | **0.97** | 1744 |
| EEG midline beta | 0.44 | <0.0001 | **0.61** | 8696 |
| B2HF (midline) | 0.99 | <0.0001 | **0.997** | 8312 |
| B2LF (midline) | 0.99 | <0.0001 | **0.997** | 8312 |
| HF2B (midline) | 0.97 | <0.0001 | **0.98** | 8560 |
| LF2B (midline) | 0.95 | <0.0001 | **0.98** | 8560 |
| **Zen Garden, unresponsive participants** | | | | |
| LF | 0.85 | <0.0001 | **0.92** | 1198 |
| HF | 0.75 | <0.0001 | **0.86** | 1198 |
| LF/HF | 0.91 | <0.0001 | **0.95** | 1198 |
| EEG midline beta | 0.49 | <0.0001 | **0.66** | 5978 |
| B2HF (midline) | 0.997 | <0.0001 | **0.998** | 5714 |
| B2LF (midline) | 0.99 | <0.0001 | **0.996** | 5714 |
| HF2B (midline) | 0.98 | <0.0001 | **0.99** | 5885 |
| LF2B (midline) | 0.95 | <0.0001 | **0.97** | 5885 |

**Table S2. Statistical summary of reliability calculations for the control cities condition.**

| Physiological metric | Correlation coefficient | Correlation p-value | **Estimated reliability** | Number of samples per half |
| --- | --- | --- | --- | --- |
| **Control cities, all participants** | | | | |
| LF | 0.80 | <0.0001 | **0.89** | 2997 |
| HF | 0.66 | <0.0001 | **0.79** | 2997 |
| LF/HF | 0.94 | <0.0001 | **0.96** | 2997 |
| EEG midline beta | 0.44 | <0.0001 | **0.61** | 15849 |
| B2HF (midline) | 0.997 | <0.0001 | **0.998** | 15488 |
| B2LF (midline) | 0.99 | <0.0001 | **0.997** | 15488 |
| HF2B (midline) | 0.98 | <0.0001 | **0.99** | 15646 |
| LF2B (midline) | 0.95 | <0.0001 | **0.97** | 15646 |
| **Control cities, responsive participants** | | | | |
| LF | 0.80 | <0.0001 | **0.89** | 1776 |
| HF | 0.61 | <0.0001 | **0.76** | 1776 |
| LF/HF | 0.94 | <0.0001 | **0.97** | 1776 |
| EEG midline beta | 0.41 | <0.0001 | **0.58** | 9392 |
| B2HF (midline) | 0.996 | <0.0001 | **0.998** | 9196 |
| B2LF (midline) | 0.99 | <0.0001 | **0.997** | 9196 |
| HF2B (midline) | 0.98 | <0.0001 | **0.99** | 9272 |
| LF2B (midline) | 0.95 | <0.0001 | **0.97** | 9272 |
| **Control cities, unresponsive participants** | | | | |
| LF | 0.81 | <0.0001 | **0.90** | 1221 |
| HF | 0.71 | <0.0001 | **0.83** | 1221 |
| LF/HF | 0.91 | <0.0001 | **0.95** | 1221 |
| EEG midline beta | 0.47 | <0.0001 | **0.64** | 6457 |
| B2HF (midline) | 0.998 | <0.0001 | **0.999** | 6292 |
| B2LF (midline) | 0.99 | <0.0001 | **0.996** | 6292 |
| HF2B (midline) | 0.98 | <0.0001 | **0.99** | 6374 |
| LF2B (midline) | 0.95 | <0.0001 | **0.97** | 6374 |

**Response magnitude to the control condition**

To verify the non-specific effect of the control condition, response magnitude to the control condition was calculated as $Response magnitude control= \frac{(STAI-Y1 before control - STAI-Y1 after control)}{STAI-Y1 baseline}$ . Linear regression analyses were performed for ECG metrics (LF, HF, LF/HF ratio), EEG (midline beta and midline alpha), and brain-heart connectivity coefficients (B2HF, B2LF, HF2B, LF2B). Results were non-significant across physiological measures: ECG (p=0.94 for LF, p=0.20 for HF, p=0.64 for LF/HF ratio), EEG (p=0.54 for midline beta, p=0.68 for midline alpha) and brain-heart connectivity coefficients (p=0.33 for B2HF, p=0.72 for B2LF, p=0.44 for HF2B, p=0.81 for LF2B). suggesting no or random effect of control condition on physiological metrics. These suggest no systematic effect of the control condition on physiological metrics.

Figure S11. Snapshots of the virtual *Zen Garden* environments.

Table S3. Timestamps and scripts used in the *Zen Garden* virtual environment.

| Timestamp | Script |
| --- | --- |
| 00:02:40 | Let my voice guide you |
| 00:02:51 | Allow yourself to be lulled by the words, sounds, images, and all the pleasant sensations… |
| 00:03:02 | If your eyelids feel heavy, feel free to let your eyes close, and you can always return to this journey whenever you like |
| 00:03:32 | In the early morning, nature comes alive with energy |
| 00:03:53 | You can feel the warmth of the sun on your shoulders |
| 00:04:13 | The water sparkles and creates a gentle rhythm that matches your steps |
| 00:04:33 | You think to yourself: I’m moving forward with energy now |
| 00:05:05 | A harmonious interplay between the wind rustling through the leaves |
| 00:05:16 | The energy of the earth and the rock |
| 00:05:25 | The gentle ripples on the surface of the lake |
| 00:05:44 | You move forward slowly, in harmony with your surroundings |
| 00:06:04 | You feel increasingly calm |
| 00:06:22 | Your gaze is captivated |
| 00:06:34 | You feel as though you’re part of a peaceful world |
| 00:07:02 | Back in the garden, a presence steps out of the shadows |
| 00:07:12 | A deer approaches |
| 00:07:31 | Calm and serene |
| 00:08:02 | You think: I feel safe |
| 00:08:30 | The bridge |
| 00:08:40 | The refreshing droplets of water on your face |
| 00:08:58 | You admire the sparkling spring as it flows and meanders through the garden |
| 00:09:12 | Among the ferns and rocks |
| 00:09:33 | Your gaze takes in the rock extending above the lake |
| 00:09:45 | You observe every detail of this beautiful landscape |
| 00:09:55 | The soft scent of water lilies fills the air |
| 00:10:15 | Breathe deeply |
| 00:10:43 | The temple is ready to welcome you |
| 00:11:03 | Clouds drift silently across the blue sky |
| 00:11:22 | Your steps take you from smooth rock to soft grass |
| 00:11:50 | Mist blends the lake and the sky |
| 00:12:09 | You continue confidently through the garden |
| 00:12:20 | You think: I walk and breathe with ease |
| 00:12:48 | You follow a path leading to the main temple |
| 00:12:59 | In the shade of pagodas, sheltered from the breeze |
| 00:13:18 | The area is lush with plants in shades of red and green |
| 00:13:48 | The rock shows tones of gray and brown, reflecting the dancing shadows of the trees |
| 00:14:03 | You walk towards the temple under the warm sun |
| 00:14:27 | You sit on a eucalyptus bench, surrounded by its soothing aroma |
| 00:14:49 | You take time to appreciate the harmony of the elements |
| 00:14:57 | The meeting of water, earth, and sky |
| 00:15:15 | You think: I savor all this beauty |
| 00:15:29 | Walking carefully along the path by the rocks |
| 00:15:43 | Your breath feels refreshed by the mist from the lake |
| 00:15:59 | Your feet return to the garden’s grass |
| 00:16:17 | Breathe, as the surface of the pool gently ripples |
| 00:16:38 | From the treetops, the silent flight of butterflies accompanies you |
| 00:17:19 | The path is easy |
| 00:17:30 | Your steps are light |
| 00:18:17 | The door to time opens, inviting you inside |
| 00:18:50 | Welcome |
| 00:19:04 | You can sit now and simply be |
| 00:19:39 | You’re surrounded by a deep sense of harmony |
| 00:19:49 | You feel calm and at peace |
